# Rab7-Mediated Endocytosis Establishes Patterning of Wnt Activity through Inactivation of Dkk Antagonism

**DOI:** 10.1016/j.celrep.2020.107733

**Published:** 2020-06-09

**Authors:** Nobuyuki Kawamura, Katsuyoshi Takaoka, Hiroshi Hamada, Anna-Katerina Hadjantonakis, Ge-Hong Sun-Wada, Yoh Wada

**Affiliations:** 1 Department of Biochemistry, Faculty of Pharmaceutical Sciences, Doshisha Women’s College of Liberal Arts, Kohdo, Kyotanabe, Kyoto 610-0395, Japan; 2 Institute of Advanced Medical Sciences, Tokushima University, 3-18-15 Kuramoto, Tokushima 770-8503, Japan; 3 RIKEN Center for Biosystems Dynamics Research, 2-2-3 Minatojima-minamimachi, Chuo-ku, Kobe 650-0047, Japan; 4 Developmental Genetics Group, Graduate School of Frontier Biosciences, Osaka University, 1-3 Yamada-oka, Suita, Osaka 565-0871, Japan; 5 Developmental Biology Program, Sloan Kettering Institute, Memorial Sloan Kettering Cancer Center, New York, NY 10065, USA; 6 Division of Biological Sciences, Institute of Scientific and Industrial Research, Osaka University, Mihogaoka 8-1, Ibaraki, Osaka 567-0047, Japan; 7 Lead Contact

## Abstract

Endocytosis has been proposed to modulate cell signaling activities. However, the role of endocytosis in embryogenesis, which requires coordination of multiple signaling inputs, has remained less understood. We previously showed that mouse embryos lacking a small guanosine triphosphate (GTP)-binding protein Rab7 implicated in endocytic flow are defective in gastrulation. Here, we investigate how subcellular defects associated with Rab7 deficiency are related to the observed developmental defects. Rab7-deficient embryos fail to organize mesodermal tissues due to defects in Wnt-β-catenin signaling. Visceral endoderm (VE)-specific ablation of *Rab7* results in patterning defects similar to systemic *Rab7* deletion. Rab7 mutants accumulate the Wnt antagonist Dkk1 in the extracellular space and in intracellular compartments throughout the VE epithelium. These data indicate that Rab7-dependent endocytosis regulates the concentration and availability of extracellular Dkk1, thereby relieving the epiblast of antagonism. This intercellular mechanism therefore organizes distinct spatiotemporal patterns of canonical Wnt activity during the peri-gastrulation stages of embryonic development.

## In Brief

Kawamura et al. find that the endocytic regulator Rab7 is required for gastrulation in mouse embryos. Rab7-dependent endocytosis participates in Wnt signaling activity by removal of the antagonist Dkk1. This study demonstrates that the endocytic pathway is essential for spatiotemporal patterning of Wnt signaling and plays a role in mammalian development.

## Graphical Abstract

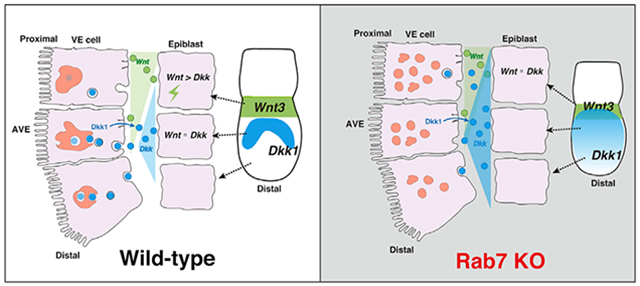

## INTRODUCTION

The architecture of multicellular organisms is established through a highly complex process in which various cellular activities are orchestrated responding to a specific context, namely the position of a cell within a population and the timing of tissue morphogenesis. Each cell maintains this spatial and temporal information by organizing its plasma membrane and extracellular environments through secretory and endocytic systems. The secretory system constitutes a synthetic route for trafficking cell surface and extracellular molecules and thus plays an indispensable role in not only assembling but also defining the extracellular milieu. The endocytic pathway participates in the retrieval of surface and extracellular components. The combination of both secretory and endocytic pathways is essential for rapid and efficient reorganization of extra- as well as intracellular activities, which are prerequisite for the execution of the morphogenetic program (Wada and Sun-Wada, 2013; Wada et al., 2016). Various membrane-anchored cell surface receptors are internalized through endocytosis and subsequently relocated to early endosomes, where receptor/ligand complexes relay signals to intracellular mediators (Barbieri et al., 2016). Thereafter, signaling complexes are either recycled back to the plasma membrane or transported to late endocytic compartments for degradation. This endocytic flow of signaling components is thought to be responsible for the spatial downregulation of distinct signal activities (Miaczynska et al., 2004; Willnow et al., 2012).

The anterior-posterior (A-P) body axis of vertebrates is established through coordinate orchestration of multiple signaling cascades, including the transforming growth factor-β (TGF-β)/Nodal, bone morphogenetic protein (BMP), fibroblast growth factor (FGF), and Wnt pathways (Muñoz-Descalzo et al., 2015; Takaoka et al., 2007; Tam and Loebel, 2007). At embryonic day 5.25 (E5.25) (corresponding to pre-gastrulation in the mouse), the most distal position of the visceral endoderm (DVE) starts forming the anterior visceral endoderm (AVE) on one side of the epiblast. The AVE secretes various signaling molecules, including Cer1 and Lefty1, which antagonize TGF-β/Nodal signaling. Consequently, the activation of Nodal signaling is restricted to the posterior side of the epiblast, where gastrulation movements occur and mesoderm is induced (Takaoka et al., 2006; Yamamoto et al., 2004). The AVE also participates in patterning of canonical Wnt signaling activities by producing the Dickkopf (Dkk) antagonists (Kimura-Yoshida et al., 2005). Dkk proteins were first identified due to their head-inducing activities in frogs (Glinka et al., 1998) and are distributed widely among vertebrates. The mechanism of how Dkk1 antagonizes signaling is unique. Dkk1 binds to the Kremen1/2 transmembrane protein, as well as the Wnt co-receptor Lrp5/6, to form a complex at the plasma membrane (Mao et al., 2002). This protein complex is dormant in terms of signaling activity and is subjected to internalization by endocytosis. Thus, Dkk1 activity results in the relocalization of essential components of the Wnt receptor complex from the plasma membrane to endocytic compartments, thereby reducing the availability of Lrp5/6 co-receptors (Sakane et al., 2010; Yamamoto et al., 2008).

Dkk1 transcripts exhibit a unique pattern of expression in peri-gastrulation embryos: they are specifically expressed at the leading edge of the AVE but absent in the cells behind the leading edge (Kemp et al., 2005; Kimura-Yoshida et al., 2005). Interestingly, both Dkks mRNA and protein exhibit this horseshoe-like distribution (Hoshino et al., 2015). This concurrence of mRNA and protein localization suggests that Dkk1 protein has a relatively short half-life. However, the underlying mechanisms regulating Dkk1 turnover remain unclear. Because Dkk1 is a secreted protein, we hypothesized endocytic relocation as a potential scenario for the negative regulation of Dkk1 antagonism.

Trafficking along the endocytic pathway is regulated by numerous molecules, which are involved in vesicle budding, tethering to the destined compartments, and membrane fusion (Hong and Lev, 2014; Spang, 2016). Rab guanosine triphosphatases (GTPases) are small GTP-binding proteins intimately involved in the regulation of membrane dynamics. The transition from early endosomes to late endosomes is regulated by Rab7 (Poteryaev et al., 2010).

Rab7 function is required for the assembly of a unique organelle, an apical vacuole within the VE of mouse embryos (Kawamura et al., 2012; Wada, 2013). Furthermore, mutant embryos lacking Rab7 are unable to develop beyond the gastrulation stage. In this study, we focused on corroborating the mechanistic link between intracellular activities, such as endocytic transport, and the establishment of higher tissue architecture of gastrulae. We found that Rab7-deficient embryos were defective in endocytic clearance of Wnt antagonist Dkk1 and thereby failed to establish correct spatial patterning of Wnt activity.

## RESULTS

### Rab7 Is Required for Canonical Wnt Signaling

**Figure 1.**
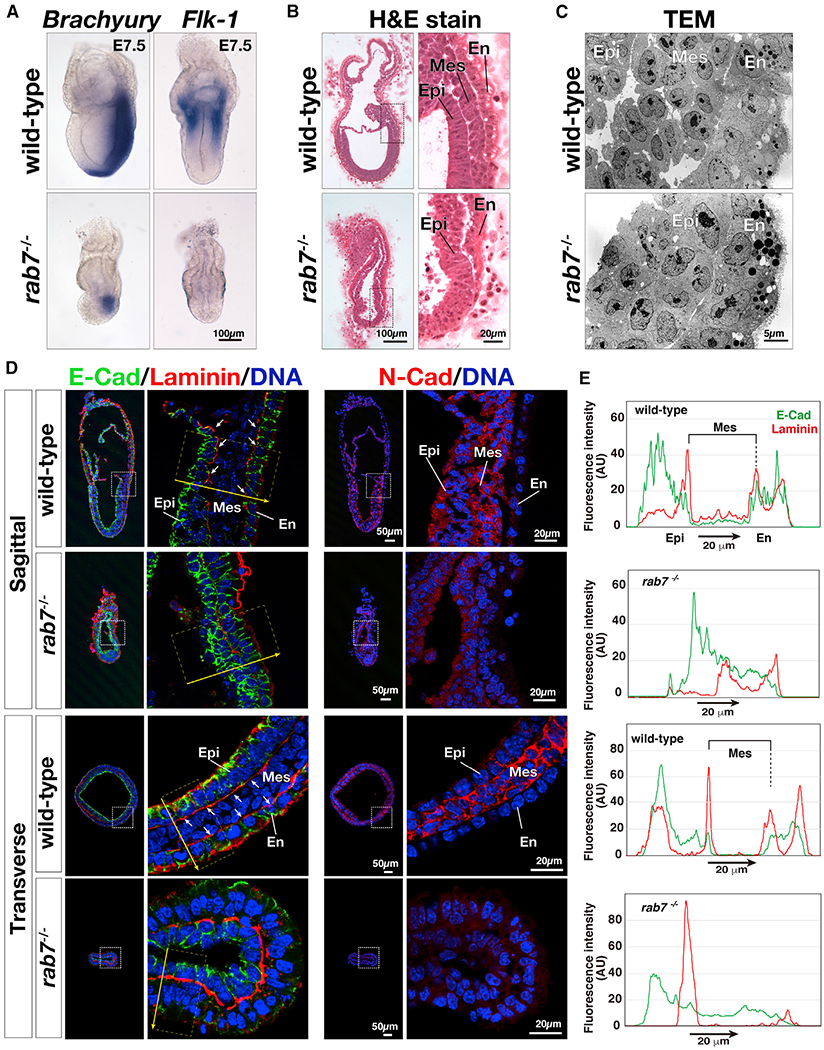
Gastrulation in Rab7-Deficient Mouse Embryos (A) Mesoderm markers *Brachyury* (anterior on left; posterior on right; wild type: n = 6; *rab7*^−/−^: n = 9) and *Flk-1* (the embryos are shown in frontal view; wild type: n = 2; *rab7*^−/−^: n = 6) were visualized by *in situ* hybridization. (B) Technovit-7100-embedded E7.5 embryos were sectioned and stained with hematoxylin and eosin. Scale bar, 100 μm. Boxed areas are shown at a higher magnification. The wild-type embryo developed mesoderm (Mes) in addition to the epiblast (Epi) and endoderm (En) at this stage (n = 5), whereas the *rab7* mutant embryo did not exhibit mesodermal tissue (n = 5). (C) The Mes in the primitive streak region of E7.5 wild-type embryos (n = 3) was observed, whereas *rab7* mutant (n = 4) contained a two-layer structure formed by the Epi and the En. (D) Frozen sections of E7.75 embryos were stained for laminin and E-cadherin (8 wild-type and 11 mutant embryos) and N-cadherin (6 wild-type and 6 mutant embryos). (E) Intensities of fluorescence of in the yellow rectangle in (D).

Mouse embryos lacking the Rab7 are defective in endocytic transport and fail to assemble large apical vacuoles within the VE epithelium (Kawamura et al., 2012). Rab7-deficient embryos are also defective in gastrulation: although able to initiate differentiation of nascent mesoderm positive for *Brachyury,* mesodermal tissues fail to migrate and the primitive streak does not elongate (Figure 1A; Kawamura et al., 2012). Transcripts for *Flk-1,* a receptor tyrosine kinase normally expressed in the extraembryonic mesoderm (Yamaguchi et al., 1993), were not detected in mutant embryos, demonstrating that extraembryonic mesoderm was not properly formed (Figure 1A). *Rab7* mutant developed less obvious mesodermal tissue between the epiblast and endoderm at E7.5 (Kawamura et al., 2012; Figures 1B and 1C). In wild type, nascent mesoderm cells adjacent to primitive streak became negative for E-cadherin, an epithelial-associated cell-adhesion molecule, and started expressing N-cadherin, a mesodermal-associated cell adhesion molecule (Figure 1D, sagittal view; Radice et al., 1997). By contrast, mutant embryos expressed lower amounts of N-cadherin and showed accumulation of E-cadherin (Figure 1D). In wild-type embryos, mesoderm wings appear between the epiblast and endoderm epithelia, resulting in two obvious basal layers positive for the extracellular matrix component laminin (Figure 1D). By contrast, in mutant embryos, only a single laminin-positive layer was detected. The plot profiles of E-cadherin and laminin confirmed that E-cadherin levels were reduced in mesoderm cells sandwiched by the laminin-positive belts in wild-type embryos but distributed more continuously in mutants (Figure 1E). These observations suggest that Rab7-deficient embryos failed to undergo the epithelial-to-mesenchymal transition associated with gastrulation and were defective in organizing tissues derivative of the primitive streak, such as the mesoderm. Consequently, mutant embryos were not able to correctly execute gastrulation and died at around E8.

**Figure 2.**
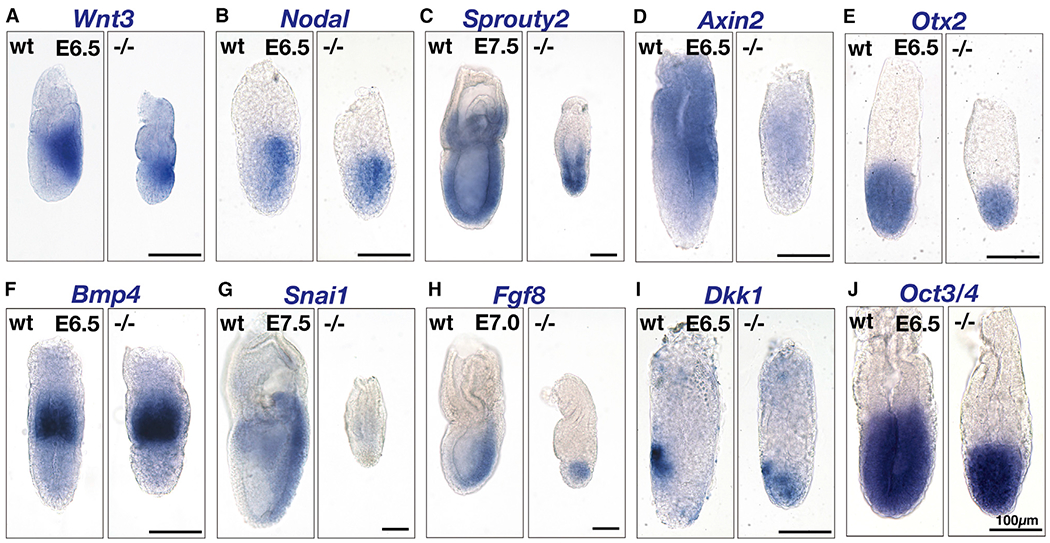
Patterning of Developmental Marker Expression in Rab7-Deficient Embryos Expression of markers was examined by *in situ* hybridization. At least 3 mutant embryos were examined for each marker. Scale bar, 100 μm. See also Figure S1 for expression patterns of *Lefty-1* and *Cer1.*

Mesoderm formation is regulated through the convergence of multiple signaling pathways, including Wnt, BMP, Nodal/TGF-β, and FGF (Arnold and Robertson, 2009; Muñoz-Descalzo et al., 2015; Rossant and Tam, 2009). Rab7-deficent embryos showed abnormal expression of genes under the regulation of the canonical Wnt pathway. In mutant, *Wnt3* was normally expressed at the proximal epiblast with a slight tilt toward the presumptive posterior side of the epiblast (Figure 2A). However, *Axin2,* a target of Wnt-β-catenin signaling (Jho et al., 2002; Lustig et al., 2002), was almost absent in mutants (Figure 2D). The expression of *Snai1* (mouse ortholog of *Drosophila* snail; Carver et al., 2001; Smith et al., 1992) as well as *Brachyury,* two genes under direct or indirect regulation of the Wnt signaling pathway (ten Berge et al., 2008), was severely reduced in mutant embryos (Figure 2G). These data collectively suggest that Rab7-deficient embryos are defective in canonical Wnt signaling.

Expression of *Brachyury* was restricted to the proximal epiblast located one side (presumable posterior side) of the epiblast of mutants (Figure 1A; Kawamura et al., 2012). A-P patterning was established at E4.5–E6.5 in mutants, as indicated by the anterior-restricted expression of *Cer1* and *Lefty-1* (Takaoka and Hamada, 2012). These two secretory proteins antagonize *Nodal,* a member of the TGF-β super family (Brennan et al., 2001; Conlon et al., 1994). In mutant embryos, expression of *Cer1* was restricted to the prospective anterior side of the E6.5 epiblast (Figure S1A). *Lefty-1* expression was variable: about a half of the mutant embryos (type I; n = 7) showed reduced expression at the anterior side, whereas in the other half (type II; n = 8), there was no detectable *Lefty-1* signal (Figure S1B).

*Nodal* was expressed in a nearly normal pattern, with a gradient along the A-P axis in mutant embryos (Figure 2B; Kawamura et al., 2012). The normal distribution of *Nodal* transcripts was in agreement with previous findings in which Wnt3 or β-catenin mutants exhibited normal *Nodal* expression at E6.0 (Ben-Haim et al., 2006; Morkel et al., 2003). The expression patterns of *Nodal* and *Cerl* showed that the A-P axis was specified in Rab7-deficient embryos (Kawamura et al., 2012). Nodal is required for the expression of Otx2, a paired-type homeodomain-containing transcription factor, which in turn is required for the establishment of A-P axis (Kimura et al., 2001; Kimura-Yoshida et al., 2005). *Otx2* was expressed normally in mutant embryos (Figure 2E). Along with the normal patterns of *Wnt3* (Figure 2A), whose expression depends on functional Nodal signaling (Ben-Haim et al., 2006), the loss of the Rab7 function had minimal impact on the Nodal signaling pathway. The epiblast marker, *Oct3/4* (aka *Pou5f1;* Rosner et al., 1990) showed well-defined epiblast localization in mutant embryos, even though the size of the epiblast was reduced when compared to wild-type embryos (Figure 2J).

FGF signaling regulates embryogenesis at multiple steps (Yamaguchi et al., 1994). FGF8 is required for the correct assembly of the primitive streak (Crossley and Martin, 1995; Sun et al., 1999). At E7.5, mutant embryos showed *Fgf8* expression in the posterior proximal region of the epiblast, although the level was lower than in wild-type embryos, reflecting defects associated with mesoderm assembly (Figure 2H). Expression of *Sprouty2,* a gene encoding a negative regulator of FGF signaling, is regulated by the FGF signaling pathway (Minowada et al., 1999). In wild-type embryos, *Sprouty2* was expressed throughout the epiblast and more abundantly in the primitive streak. This pattern of expression was conserved in mutant embryos (Figure 2C). Thus, the expression of a key FGF ligand acting at gastrulation, as well as the perception of FGF signaling, was not affected by Rab7 deficiency.

### Rab7 Function in the VE Is Essential for Gastrulation

**Figure 3.**
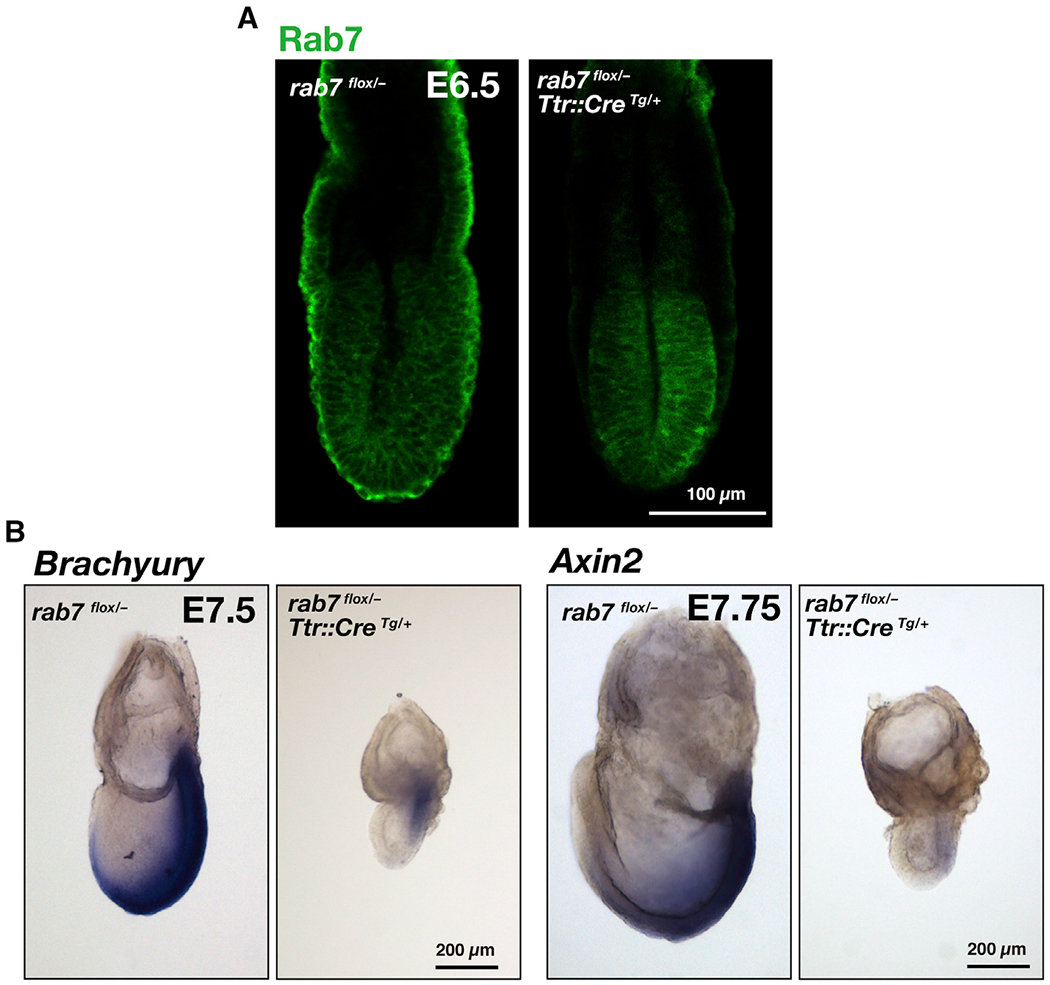
Defective Gastrulation upon VE-Specific Ablation of Rab7 Function (A) Female *rab7*^flox/flox^ mouse was crossed with a male mouse (*rab7*^+/−^, *Ttr::Cre*^Tg/+^). Embryos were dissected at E6.5, and Rab7 was detected by immunofluorescence. The rab7 heterozygote (*rab7*^flox/−^: n = 11) and rab7 VE deletion (*rab7*^flox/−^, *Ttr::Cre*^Tg/+^: n = 8) were observed. (B) *Brachyury* expression was examined in 3 rab7 heterozygotes (*rab7*^flox/−^) and 10 rab7 VE deleted (*rab7*^flox/−^, *Ttr::Cre*^Tg/ +^) embryos. *Axin2* expression was observed in 8 rab7 heterozygotes (*rab7*^flox/−^) and 9 rab7 VE deleted (*rab7*^flox/−^, *Ttr::Cre*^Tg/ +^) embryos. See also Figure S2 for phenotype of epiblast-specific ablation of Rab7 function.

Despite *Rab7* transcripts being expressed equally in the epiblast and extraembryonic ectoderm, the VE exhibits a greater abundance of Rab7 protein than the epiblast and extraembryonic ectoderm (Kawamura et al., 2012). We therefore investigated whether Rab7 function was required in the epiblast, which gives rise to the definitive germ layers (mesoderm, endoderm, and ectoderm). We used a *Ttr::Cre*^Tg/+^ strain of mice, driving expression of the Cre recombinase throughout the VE of E5.5 embryos (Kwon and Hadjantonakis, 2009). We crossed *Ttr::Cre*^Tg/+^ *rab7*^+/−^ male mice to *rab7*^flox/flox^ female mice (Kawamura et al., 2012). At E6.5, all embryos exhibited normal morphology. However, in *Ttr::Cre*^Tg/+^; *rab7*^flox/−^ embryos, Rab7 was below detectable levels in the VE, indicating VE-specific inactivation of Rab7 (Figure 3A). The gross morphology of *Ttr::Cre*^Tg/+^; *rab7*^flox/−^ embryos was similar to mutant embryos having a systemic deletion of Rab7 function. *Brachyury* expression was initiated at the proximal posterior region, but *Brachyury*-positive cells did not organize into an elongated primitive streak (Figure 3B). *Axin2* expression was significantly reduced in embryos lacking Rab7 function in the VE (Figure 3B). These observations demonstrate that Rab7 function in the VE is essential for gastrulation.

We next examined the consequence of an epiblast-specific deletion of Rab7 function by expressing the *Sox2*-promoter-driven Cre (Hayashi et al., 2002). Rab7 protein was undetectable in the epiblast of *Sox2::Cre*^Tg/+^ *rab7*^flox/−^ embryos at E6.5, demonstrating efficient ablation of Rab7 function. By contrast to the VE-specific deletion, the majority of epiblast-specific knockout embryos (4/5) did not exhibit severe developmental defects at E7.5 (Figure S2), showing that loss of Rab7 in the epiblast resulted in less severe defects than loss of the Rab7 function in the VE. Thus, although expression levels of Rab7 were high in the epiblast of wild-type embryos, its function in the epiblast was dispensable for gastrulation.

### Rab7-Dependent Endocytosis Controls Dkk Antagonism

During gastrulation, loss of Rab7 function in the VE was sufficient to result in developmental defects that were comparable to systemic loss of *Rab7.* This non-cell-autonomous aspect suggests an intercellular mechanism might be responsible for the observed developmental defects. The endocytic compartments are traditionally considered to provide a degradation mechanism. We therefore hypothesized that the defects in Wnt signal transduction may result from failure of abrogating a negative regulation on the pathway.

Wnt signaling is regulated negatively by Dkk proteins (Glinka et al., 1998; Lewis et al., 2008), whose binding to the Wnt coreceptor Lrp5/6 and Kremen leads to endocytosis and a concomitant decrease in Wnt signal receptivity at the plasma membrane (Davidson et al., 2002; Mao et al., 2002; Sakane et al., 2010; Yamamoto et al., 2008). Dkk1 shows a unique expression pattern at E6-E6.5, being restricted to the leading front of the migrating AVE (Kimura-Yoshida et al., 2005; Figure 2I). Rab7-deficient embryos expressed *Dkk1* transcripts at a comparable level to wild-type embryos. *Dkk1* expression is regulated by Otx2 (Kimura-Yoshida et al., 2005), which was expressed normally in mutant embryos, indicating that transcriptional regulation of both *Otx2* and *Dkk1* was unaffected. The *Dkk1* transcript in E6.5 mutant embryos did however exhibit a more broadened distribution (Figure 2I), most likely reflecting impaired AVE patterning. This was also the case for the expression of *Cer1* and *Lefty1* (Figure S1; Kawamura et al., 2012). This impaired patterning of AVE was also reported in embryos defective in canonical Wnt signaling (Barrow et al., 2007), supporting our view that Rab7-deficient embryos had reduced Wnt signaling activity.

**Figure 4.**
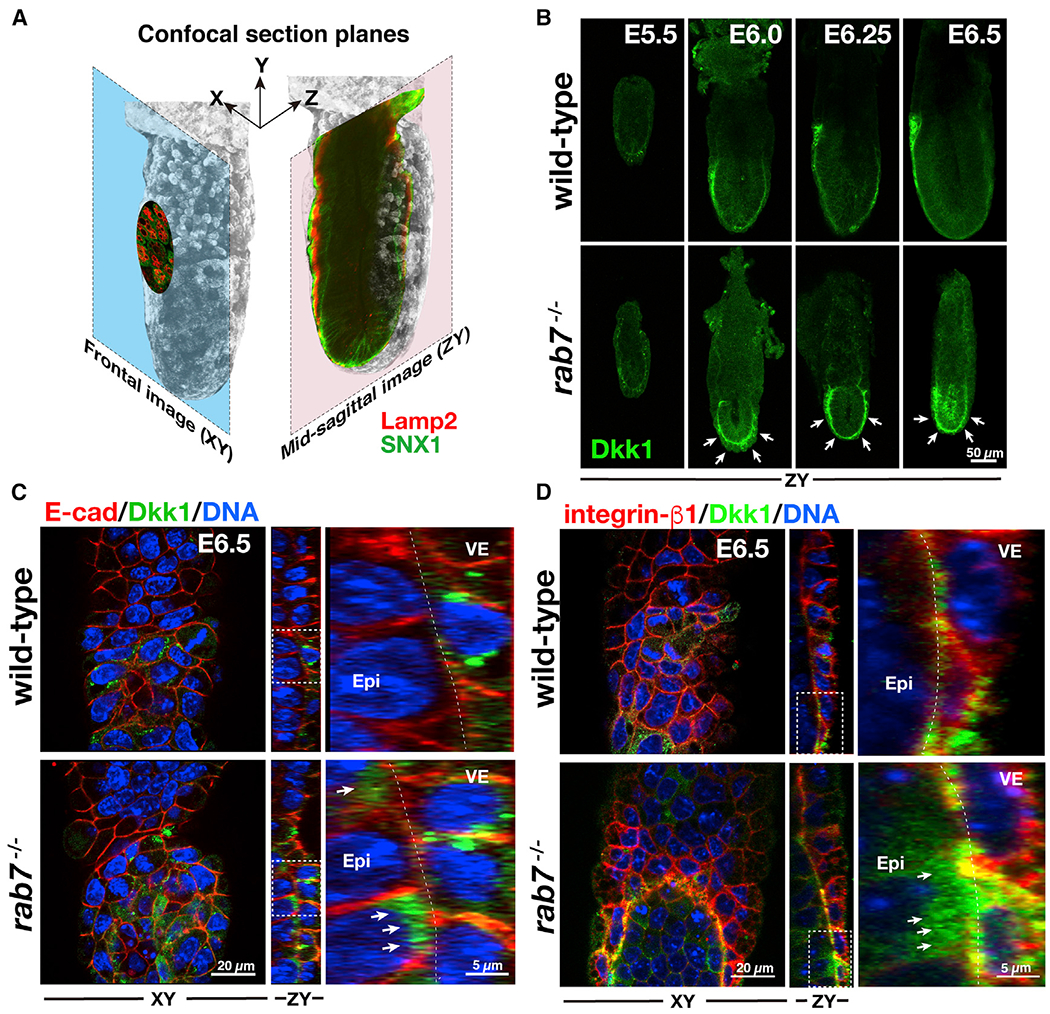
Accumulation of Wnt Antagonist, Dkk1 Proteins in Rab7-Deficient Embryos at Peri-gastrulation Stages (A) Schematic diagram of optical sections of a whole mounted embryo. (B) Localization of Dkk1 at E5.5 (wild type: n = 2 and *rab7*^−/−^: n = 5), E6.0 (wild type: n = 20 and *rab7*^−/−^ n = 16), E6.25 (wild type: n = 10 and *rab7*^−/−^: n = 10), and E6.5 (wild type: n = 13 and *rab7*^−/−^: n = 6). (C) Elevated signal of Dkk1 in Rab7-deficient embryos between the visceral endoderm (VE) and Epi. E6.5 wild-type (n = 4) and Rab7-deficient embryos (n = 4) were stained for Dkk1 (green) and E-cadherin (red). The areas outlined by white dashed lines are magnified. Arrows indicate the Dkk1 signals outside the VE cells. (D) E6.5 wild-type (n = 10) and Rab7-deficient (n = 7) embryos stained for Dkk1 (green) and integrin β1 (red). The areas outlined by white dashed lines are magnified. Arrows indicate the Dkk1 signals outside the VE cells. See also Videos S1, S2, S3, S4, S5, and S6 for three-dimensional reconstitution of Dkk1 patterning.

In E6.5 wild-type embryos, Dkk1 protein was abundant at the leading edge of the presumptive AVE (Figure 4B), exhibiting a horseshoe-like pattern (Video S1; Hoshino et al., 2015), comparable to that of the transcript (Figure 2I). In mutant embryos, Dkk1 was more abundant and the region positive for Dkk1 expanded (Figure 4B; Video S2). At E5.5, when AVE migration commences at the distal region of the epiblast, low levels of Dkk1 protein accumulation were observed in the epiblast of wild-type and mutant embryos. At this stage, no significant difference was observed in Dkk1 distribution. By E6.0, Dkk1 protein became apparent in the leading edge of the proximally migrating AVE of wild-type embryos, although the distal part of the epiblast exhibited a lower level of expression. By contrast, high levels of Dkk1 had accumulated throughout the entire embryonic part of mutant embryos. These elevated levels of Dkk1 were evident until E6.5. At E6.5, Dkk1 protein localization was predominantly inside VE cells of wild-type embryos but undetectable in the extracellular space between VE and the neighboring epiblast (Video S3; Figure 4B). However, mutant embryos exhibited elevated levels of Dkk1 protein, which had accumulated in the extracellular space (Video S4; Figure 4B).

We labeled Dkk1 together with E-cadherin, which is localized at the basolateral membrane of epithelial cells. The majority of Dkk1 signal was observed within VE cells (Figure 4C). By contrast, in mutant, strong Dkk1 accumulation was observed in the border of epiblast and VE. In addition, a few puncta of Dkk1 were observed at the lateral extremity of VE cells (Figure 4C). Some of the Dkk1 protein was localized in close proximity to integrin β1 (Figure 4D; Videos S5 and S6), which marks the basal cell surface comprising adherens junctions (Shafaq-Zadah et al., 2016). It therefore appeared as if a significant amount of Dkk1 had accumulated in the extracellular space of Rab7-deficient embryos.

**Figure 5.**
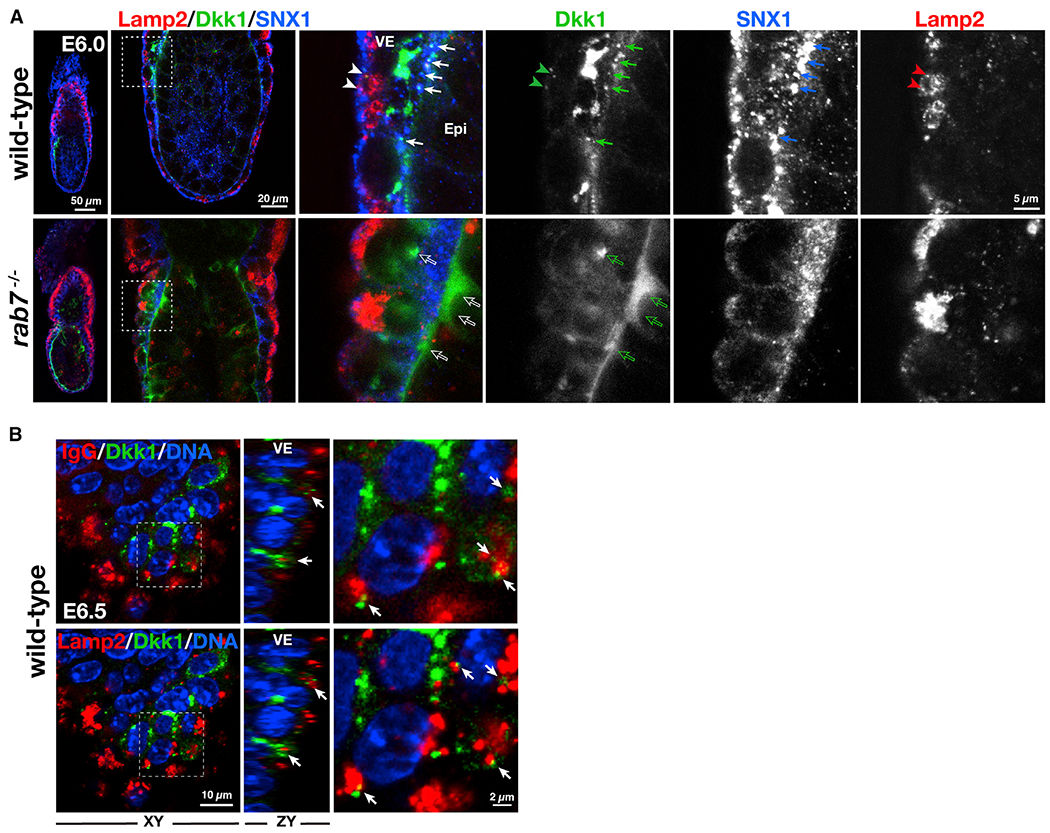
Rab7-Dependent Trafficking of Dkk1 Creates Wnt Signal Pattern in Peri-gastrulation Embryo (A) E6.0wild-type (n = 20) and Rab7-deficient embryos (n = 16) stained for Dkk1 (green) and sorting nexin 1 (SNX1, red). Arrows indicate colocalization of Dkk1 and SNX1 in the wild-type embryos. Outlined arrows indicate Dkk1 signals observed in Rab7 mutants, which colocalizes neither SNX1 nor Lamp2. Single-channel images are also shown. (B) E6.5 wild-type embryos (n = 21) stained for Dkk1 (green), Lamp2 (red), and mouse IgG (red). Arrows indicatethe Dkk1 signals overlapping with IgG or Lamp2. See also Videos S7 and S8.

The intracellular Dkk1 signals overlapped with sorting nexin 1 (SNX1), an early endosome marker, within VE cells (Figure 5A), suggesting that Dkk1 was internalized by endocytosis. SNX1-positive compartments were distributed broadly in Rab7-deficient cells, reflecting trafficking defects in the endocytic pathway (Kawamura et al., 2012). Colocalization of Dkk1 with SNX1 was absent in mutant embryos, and Dkk1 proteins appeared to accumulate outside the basal membrane of VE cells (Figure 5A). Dkk1 and Rab4, a resident of subdomains of early endosomes (Daro et al., 1996), existed in close proximity in wild-type VE cells. By contrast, Dkk1 and Rab4 were separated in mutant embryos (Figure S3). These observations suggest that defects in Dkk1 trafficking occur at endocytic compartments rather than in the secretory pathway, leading to accumulation of Dkk1 in extracellular spaces.

The VE epithelium takes up immunoglobulin by endocytosis, where it is delivered to Lamp2-positive apical vacuoles (Aoyama et al., 2012; Kawamura et al., 2012). Some internalized Dkk1 at the apical side of VE cells were positive for Lamp2 and immunoglobulins (Figures 5A and 5B), indicating that internalized Dkk1 was being delivered to the terminal compartment of the endocytic pathway, where presumably it is destined for degradation (Videos S7 and S8). By contrast, in Rab7-deficient VE cells, Lamp-2 compartments, which are highly fragmented, did not contain Dkk1 (Figure 5A, open arrows), suggesting that lysosomal trafficking of Dkk1 was defective.

Within the VE, endocytic flow occurs via microautophagy, whereby endosomes are engulfed by apical vacuoles without membrane fusion. The endosomal membranes are then disrupted inside the apical vacuole by lipase activity (Kawamura et al., 2012). In this process, endocytosed materials transiently appear within apical vacuoles as distinctive compartments, reflecting an engulfed but neither merged nor digested status. We often observed Dkk signals at close proximity to Lamp2- or immunoglobulin-positive apical vacuoles as distinctive spots (Videos S7 and S8). This suggests that microautophagy participated, at least in part, in the trafficking of Dkk1 to apical vacuoles.

## DISCUSSION

The relevance of endocytosis in mammalian embryogenesis has been demonstrated by a number of studies, implicating it in nutritional and signal transduction (Aoyama et al., 2012; Kawamura et al., 2012; Perea-Gomez et al., 2019; Shim et al., 2006; Strope et al., 2004; Takasuga et al., 2013; Zheng et al., 2006). Here, we demonstrate that Rab7 is required for the proper patterning of Wnt signaling activity by removal of the Wnt antagonist Dkk1. This endocytic retrieval and compartmentalization of Wnt antagonist(s) constitutes an essential mechanism for establishing embryonic patterning during peri-gastrulation development of the mouse embryo.

Stabilization of β-catenin, a cytosolic signal transducer for the Wnt signaling pathway, involves sequestration of GSK3 protein kinase into late endosomes (Albrecht et al., 2018, 2019; Taelman et al., 2010). Wnt signaling has been shown to stabilize the microphtalmia-associated transcription factor (MITF), which regulates the expression of genes required for endosome/lysosome biogenesis (Ploper et al., 2015). These findings demonstrate that Wnt signaling and endosome/lysosome dynamics are intimately connected via a cell-autonomous mechanism. Here, we demonstrate a non-cell-autonomous mechanism for endosomal regulation of Wnt signaling in gastrulating embryos. Rab7 function in the epiblast is not required for gastrulation. However, loss of Rab7 in the VE causes severe gastrulation defects. Notably, β-catenin function has been shown to be required in the embryonic ectoderm, but not in the extraembryonic tissues, for primitive streak development (Engert et al., 2013; Huelsken et al., 2000; Lickert et al., 2002). The contrasting requirements for Rab7 and β-catenin suggest that they act at different steps within the signaling cascade.

Soon after the mouse embryo establishes a cup-shaped “egg cylinder” epiblast (E5.25), anterior markers localized within the adjacent VE tissue layer become highly expressed at the region adjacent to the most distal pole of the epiblast, demarcating the DVE (Takaoka et al., 2007). During subsequent stages of development, the *Lefty-1*- and *Cer1*-positive initially distal region extends toward a more proximal region of the epiblast along one side (demarcating the future anterior side of the embryo, being referred to as the AVE). *Lefty-1* and *Cer1* are expressed throughout the migrating AVE population (Perea-Gomez et al., 2002). By contrast, *Dkk1* transcripts demarcate a subpopulation of the AVE as they exhibit a unique horseshoe-like pattern, corresponding to the frontal ridge of the AVE (Kimura-Yoshida et al., 2005). The expression of *Dkk1* is under regulation of canonical Wnt signaling (González-Sancho et al., 2005; Niida et al., 2004), and Wnt3-deficient embryos fail to express *Dkk1* in the AVE (Lewis et al., 2008). Therefore, the most proximal leading edge of the AVE, highly influenced by Wnt ligands expressed in the proximal epiblast, exhibits a high level of *Dkk1* expression. However, Dkk1 protein shuts off Wnt in the trailing (more distally located) cells, resulting in a reduction of *Dkk1* transcription. This feedback loop results in the distinctive localization of *Dkk1,* resulting in a horseshoe-like distribution.

Once *Dkk1* acquires its unique pattern, an additional layer of regulation impacts the dynamics of the Dkk1 protein. Fluorescent protein (EGFP) reporters expressed from the *Dkk1* locus are localized throughout the VE, failing to recapitulate the distribution of endogenous Dkk1 as observed at the mRNA and protein level. However, when EGFP is destabilized by tagging it with degradation signals, it reproduces the pattern of Dkk1 protein, suggesting that the distribution of Dkk1 is highly dependent on protein turnover (Hoshino et al., 2015). Because Dkk1 is a secreted protein, the active silencing mechanism that physically degrades it must exploit an extracellular and/or endocytic mechanism and be distinct from those for cytosolic degEGFP.

We propose that the aberrant Wnt patterning was causative for the developmental defects associated with the loss of Rab7 function. *Lefty-1* expression becomes undetectable in the E6.0 β-catenin mutant (Morkel et al., 2003), whereas less perturbed expression of *Cerl* was associated with various mutations of Wnt components (Hsieh et al., 2003; Liu et al., 1999; Morkel et al., 2003). Rab7 deficiency brought similar differential impacts on *Cerl* and *Lefty-1,* supporting that the Rab7 function is rather selective in canonical Wnt pathway. Rab7 mutant showed an altered patterning in the AVE markers. Loss of either Wnt3 or β-catenin leads broaden AVE, marked by *Dkk1* and *Cerl* (Barrow et al., 2007), similarly to what occurred in the Rab7-deficient embryos. These observations are coherent that Wnt signaling was affected. However, we are not able to rigorously exclude possibilities that Nodal and other signaling pathways are also under regulation of Rab7-dependent endocytosis and their dysregulation is also participated in the embryonic phenotype.

The VE of peri-gastrulation embryos exhibits a unique membrane process, microautophagy, whereby large apical vacuoles engulf endosomes at late stages of endocytosis (Wada et al., 2013). The results we present here demonstrate that Rab7-mediated microautophagy in the VE cells plays a role in Wnt signaling at peri-gastrulation stages. Our observations suggest that microautophagy is not only required for nutritional supplement but also implicated in the spatial regulation of signaling.

## STAR METHODS

### RESOURCE AVAILABILITY

#### Lead Contact

Further information and requests for resources and reagents should be directed to and will be fulfilled by the Lead Contact, Yoh Wada (yohwada@sanken.osaka-u.ac.jp)

#### Materials Availability

The mutant and Tg mice generated in this study will be made available on request from the Lead Contact, but we may require a shipping/handling payment and/or a completed Materials Transfer Agreement.

#### Data and Code Availability

The published article includes all data generated during the study and the raw data is available on request from the Lead Contact. No code was developed for this study.

### EXPERIMENTAL MODEL AND SUBJECT DETAILS

*Rab7* mutant mice (RRID:IMSR_RBRC05600 and RRID:IMSR_RBRC05601) were described previously (Kawamura et al., 2012). *Ttr::Cre* transgenic mice were generated by injecting a Ttr-Cre construct (AddGene 32606)(Kwon and Hadjantonakis, 2009) into pronucleus of E0.5 embryos obtained from a hybrid strain of C57BL/6J and C3H/HeJ. The *Sox2::Cre* mouse (RRID:IMSR_HAR:3359) (Hayashi et al., 2002) were obtained from The Jackson Laboratory. C57BL/6 and ICR mice were purchased from SLC Japan. Adult female mice (> 6 wks) were placed with males (> 10 wks) and examined daily for the presence of a copulation plug. Embryos were obtained from pregnant females sacrificed by isoflurane anesthesia followed by *in vivo* perfusion fixation, then processed for immunostaining or whole mount *in situ* hybridization as described previously (Aoyama et al., 2012; Kawamura et al., 2012; Yamamoto et al., 2001). Embryos were staged according to the dissection time (noon of the vaginal plug as E0.5) and morphology. All animal experiments were approved by the institutional committees (ISIR, Osaka Univ. and Doshisha Women’s College), and were carried out in accordance with the rules and regulations of the institutions and the government.

### METHOD DETAILS

#### Genotyping

Mouse genotypes were determined by PCR amplification. After morphological observation and recording, embryos were incubated in 20 μL (for E6.5 and earlier) or 40 μL (for E7.5) of QuickExtract solution (Epicenter) at 50°C overnight and heat inactivated at 95°C for 15 min. One microliter of embryo lysate was used for a PCR reaction of 20 μL volume. PCR products were analyzed either by agarosegel electrophoresis or EvaGreen fluorescence in real-time PCR detection systems (Applied Biosystems 7000 or TaKaRa TP950). The primers used for genotyping were listed in the Key Resources Table. The wild-type allele of *Rab7* (*Rab7*^+^) was detected as 0.94-kb and 0.32-kb PCR products with primer pairs of Rab7-S10/Rab7-A02 and Rab7-S15/Rab7-A16, respectively. *rab7*^−^ was detected as 0.45-kb product with a Rab7-S02/Rab7-A05 primer pair. *Rab7*^flox^ allele gave signals of 1.0-kb and 0.83-kb with primer pairs Rab7-S10/Rab7-A02 and Rab7-S15/Rab7-A16, respectively. The presence of *Ttr::Cre* or *Sox2::Cre* transgene was determined by PCR signals of a 0.37-kb fragment. A schematic presentation of the wild-type and modified versions of the *Rab7* locus was reported previously (Kawamura et al., 2012).

#### Technovit Section and Histological Analysis

Mouse embryos were dissected at E7.5 and embedded in Technovit 7100 resin. Sagittal sections were stained with hematoxylin and eosin.

#### Immunofluorescence Staining

Immunofluorescence staining was performed as previously described (Kawamura et al., 2012). For immunohistochemistry, fixed embryos were incubated with primary and secondary antibodies in a blocking solution containing 0.05% Tween-20, 0.5% TSA blocking reagent (PerkinElmer) and 1% normal donkey serum in PBS. Nuclear DNA was stained by TOPRO-3 or DAPI.

#### Microscopy

Immunostained embryos were immersed in VECTASHIELD (Vector Laboratories), and mounted in 0.1% gellan gum (Sigma-Aldrich) in PBS plus 40% glycerol and 0.2% Tween-20 in a glass-top 35-mm dish. Bright-field images of the histological or *in situ* hybridization specimens were acquired with an Olympus BX50 microscope and recorded using a MicroPublisher5.0 (QImaging) camera (tagged image file format mode, auto white balance). Immunofluorescence samples were viewed with a confocal laser scanning microscope (Zeiss LSM 510META or LSM800). Serial Z stacks of fluorescence images in frontal focal plane (XY) were obtained at 0.92 μm intervals by confocal microscopy. The sagittal (ZY) images were converted using Zeiss ZEN2.5 lite software. For electron microscopy, embryos were fixed in 2.5% glutaraldehyde and 4% PFA in 0.1 M potassium phosphate buffer (pH 7.4), and then processed using standard procedures (Sun-Wada et al., 2000, 2006). Electron microscopic observations were conducted at the Hanaichi Ultra-Structure Research Institute (Okazaki, Japan).

### QUANTIFICATION AND STATISTICAL ANALYSIS

The sample size of the experiments was defined based on previous experimental experience. For treatments, the embryos were allocated randomly to treatments, but the investigators were not blinded to allocation. Sample numbers of each genotype can be found in the Figure Legends, whereby n values stand for the number of embryos observed. Developmental maker expression in E5.5-7.5 embryos (Figure 2), we observed 7 *rab7* mutants for *Wnt3,* 3 for *Nodal,* 5 for *Sprouty2,* 7 for *Axin2,* 3 for *Otx2,* 6 for *Bmp4,* 4 for *Snail1,* 4 for *Fgf8,* and 8 for *Dkk1.* Intensities of fluorescence of E-cadherin and laminin in Figure 1 were analyzed using the Plot Profile module in NIH ImageJ software.

## Supplementary Material

Video 2

Video 3

Video 1

Video 4

Video 7

Video 5

Video 8

Video 6

1

## Figures and Tables

**Table:** KEY RESOURCES TABLE

REAGENT or RESOURCE	SOURCE	IDENTIFIER
Antibodies		
Rat anti-E-cadherin	Takara Bio	M108, Clone ECCD- 2
Rabbit anti-Laminin	LSL	LB-1013, RRID:AB_605066
Rabbit anti-N-cadherin	Abcam	Ab18203, RRID:AB_444317
Rabbit anti-Rab7	Prepared in our lab	Kawamura et al., 2012 https://doi.org/10.1038/ncomms2069
Goat anti-Dkk1	R&D systems	AF1765, RRID:AB_354977
Rat anti-integrin beta 1, clone MB1.2	Merck Millipore	MAB1997, RRID: AB_2128202
Rat anti-Lamp2	DSHB	Clone GL2A7, RRID:AB_2314737
Rabbit anti-SNX1	Prepared in our lab	Nakamura et al., 2001
Sheep anti- Anti Digoxigenin-AP Fab Fragments	Roche	11093274910 RRID:AB_514497
Rabbit anti-Rab4	Abcam	Ab13252 RRID:AB_2269374
Experimental Models: Organisms/Strains		
*Mus musculus*/Rab7 floxed	Kawamura et al., 2012	RRID:IMSR_RBRC0 5600
*Mus musculus*/Rab7 null	Kawamura et al., 2012	RRID:IMSR_RBRC0 5601
*Mus musculus*/Tg(Ttr-Cre)	This study	N/A
*Mus musculus*/Tg(Sox2-cre)1Amc	The Jackson Laboratory	RRID:IMSR_HAR:33 59
Oligonucleotides		
Primer Rab7-S02 ACCTGGAAGAGTGAACCAAG GGTCAGCATG	*Rab7* intron1-2, sense strand, Kawamura et al., 2012	N/A
Primer Rab7-A05 AATTTAGGATTGGGGTGTGG CTCCGTGCTC	*Rab7* intron1-2, antisense strand, Kawamura et al., 2012	N/A
Primer Rab7-S10 CAAATGGCTCATTAGTTCTTG AGCTACCAC	*Rab7* intron 1-2, sense strand, Kawamura et al., 2012	N/A
Primer Rab7-A02 ACCCCTGCCTGGGATTTTGG TCCTGGATTC	*Rab7* intron 1-2, antisense strand, Kawamura et al., 2012	N/A
Primer Rab7-S15 GGATAAAATAGCAGTAAAAG CACGGTCGGG	*Rab7* intron 3-4, sense strand, Kawamura et al., 2012	N/A
Primer Rab7-A16 GGTGGATTTTTCTGAGTTTGA GGCCAGCCT	*Rab7* intron 3-4, antisense strand, Kawamura et al., 2012	N/A
Primer Cre-Fw ACCTGAAGATGTTCGCGATTATCT	Bacteriophage P1, Cre recombinase sense strand	N/A
Primer Cre-Rv ACCGTCAGTACGTGAGATATCTT	Bacteriophage P1, Cre recombinase antisense strand	N/A
Recombinant DNA		
Ttr:Cre	Kwon and Hadjantonakis, 2009	RRID:Addgene_326 06
Software and Algorithms		
Zeiss Laser Scanning Microscope LSM510	Carl Zeiss	Version 4.2 SP1
Zeiss Laser Scanning Microscope LSM800	Carl Zeiss	ZEN 2.3
QImaging QCapture Pro	Teledyne QImaging	Version 6.0
ImageJ		https://imagej.nih.gov/ij/

## Highlights

- Rab7-deficient embryos are defective in gastrulation
- Rab7 is required in visceral endoderm for gastrulation but dispensable in epiblast
- Endocytic clearance of Dkk antagonist establishes spatial patterning of Wnt activity
