# Supplementary material for: Rab7-Mediated Endocytosis Establishes Patterning of Wnt Activity through Inactivation of Dkk Antagonism
Source: Cell Rep. Author manuscript; Available in PMC 2021 Jun 2. (PMC8171381; doi:10.1016/j.celrep.2020.107733)
Supplement: 1 [file NIHMS1703082-supplement-1.pdf]

Cell Reports, Volume *31*

Supplemental Information

Rab7-Mediated Endocytosis Establishes Patterning of Wnt Activity through Inactivation of Dkk Antagonism

Nobuyuki Kawamura, Katsuyoshi Takaoka, Hiroshi Hamada, Anna-Katerina Hadjantonakis, Ge-Hong Sun-Wada, and Yoh Wada

Supplementary Information

Rab7-mediated endocytosis establishes patterning of Wnt activity through inactivation of Dkk antagonism.

Nobuyuki Kawamura, Katsuyoshi Takaoka, Hiroshi Hamada, Anna-Katerina Hadjantonakis, Ge-Hong Sun-Wada, Yoh Wada

Inventory of Supplementary Information

Figure S1, related to Figure 2, Expression patterns of *Cer1* and *Lefty*-1.
Figure S2, related to Figure 3, Epiblast-specific deletion of Rab7
Figure S3 related to Figure 5, Dkk1 trafficking occurred at endocytic compartments.

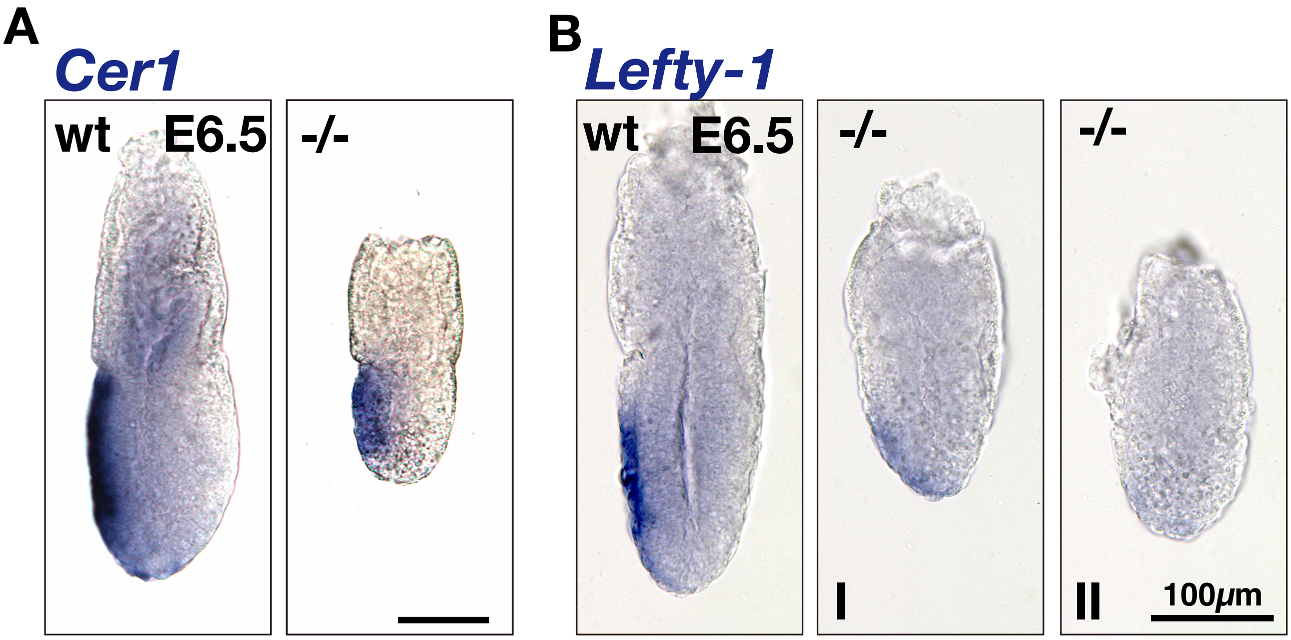

Figure S1: Patterning of *Cer1* and *Lefty-1* expression in Rab7-deficient embryos

Expression patterns of *Cer1*(**A**) and *Lefty-1*(**B**) transcripts at the indicated developmental stages were examined by *in situ* hybridization. At least 3 mutant embryos were examined for each marker molecules. Scale bar, 100 µm.

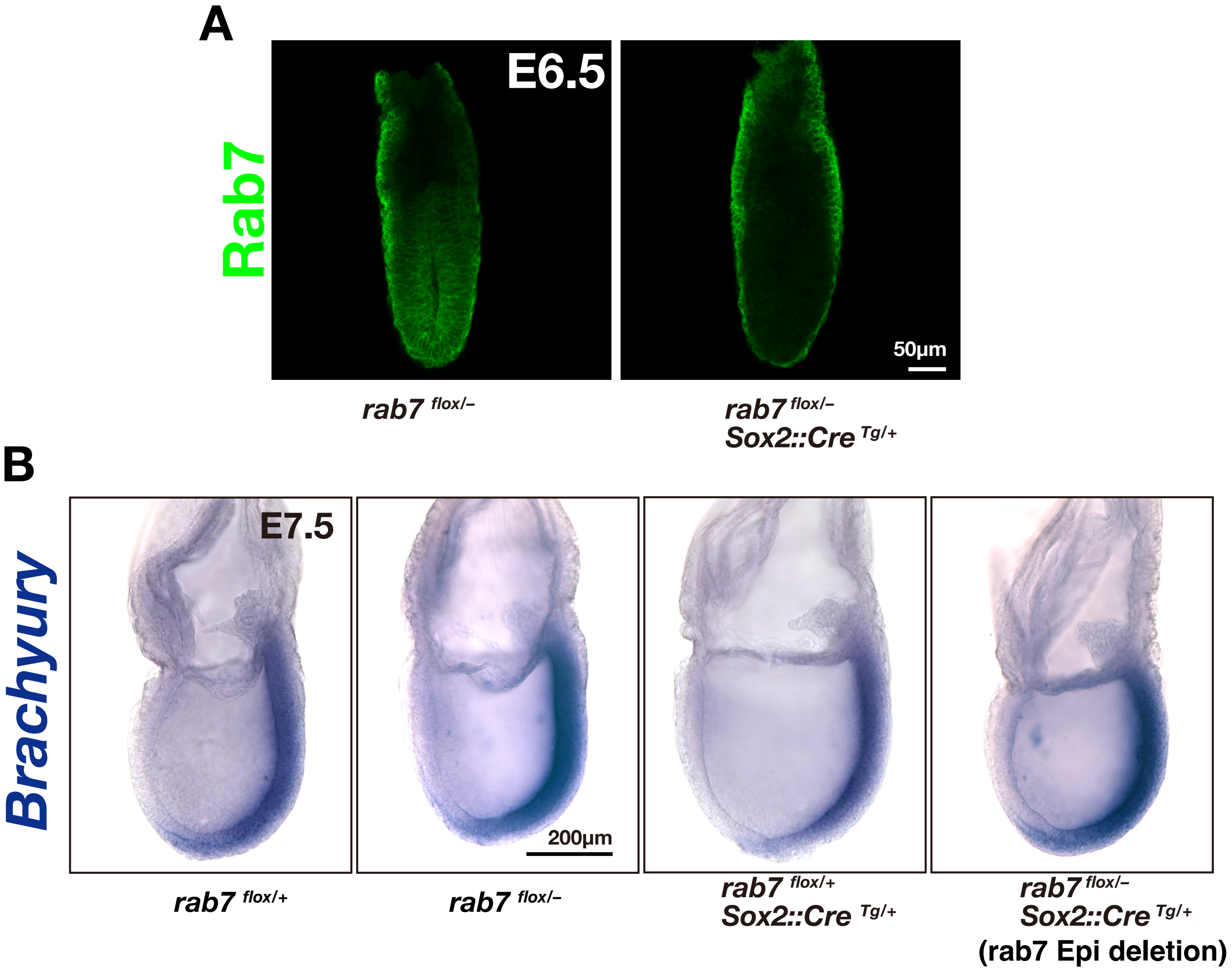

Figure S2: Epiblast-specific deletion of the Rab7-function

(**A**) A female *rab7*$^{flox/flox}$ mouse was crossed with a male mouse heterozygous for *Sox2::Cre* and *rab7* (*rab7*$^{+/-}$, *Sox2::Cre*$^{Tg/+}$)$^{-}$. Embryos were dissected at E6.5, and Rab7 was detected by immunofluorescence staining. After imaging, the presence of *rab7* wild-type allele was determined by PCR on the lysate of whole embryos, as described in Figure 3. In E6.5 *rab7*$^{flox/-}$, *Sox2::Cre*$^{Tg/+}$ embryos, Rab7 protein was under detectable levels in the epiblast (*rab7*$^{flox/+}$: 5 embryos and *rab7*$^{flox/-}$, *Sox2::Cre*$^{Tg/+}$: 5 embryos. Scale bar, 50 µm.

(**B**) Embryos were dissected at E7.5, and the *Brachyury* expression was examined by in situ hybridization. *Brachyury* expression was examined in 4 *rab7*$^{flox/+}$, *Sox2::Cre*$^{Tg/+}$, 5 *rab7*$^{flox/-}$, *Sox2::Cre*$^{Tg/+}$, 7 *rab7*$^{flox/+}$, and 3 *rab7*$^{flox/-}$ embryos. Even in the embryos lacking the Rab7 function in the epiblast, the *Brachyury* transcripts, as well as the gross morphology, appeared normal at E7.5, showing that the Rab7 function in the epiblast was dispensable for the gastrulation. Scale bar, 200 µm.

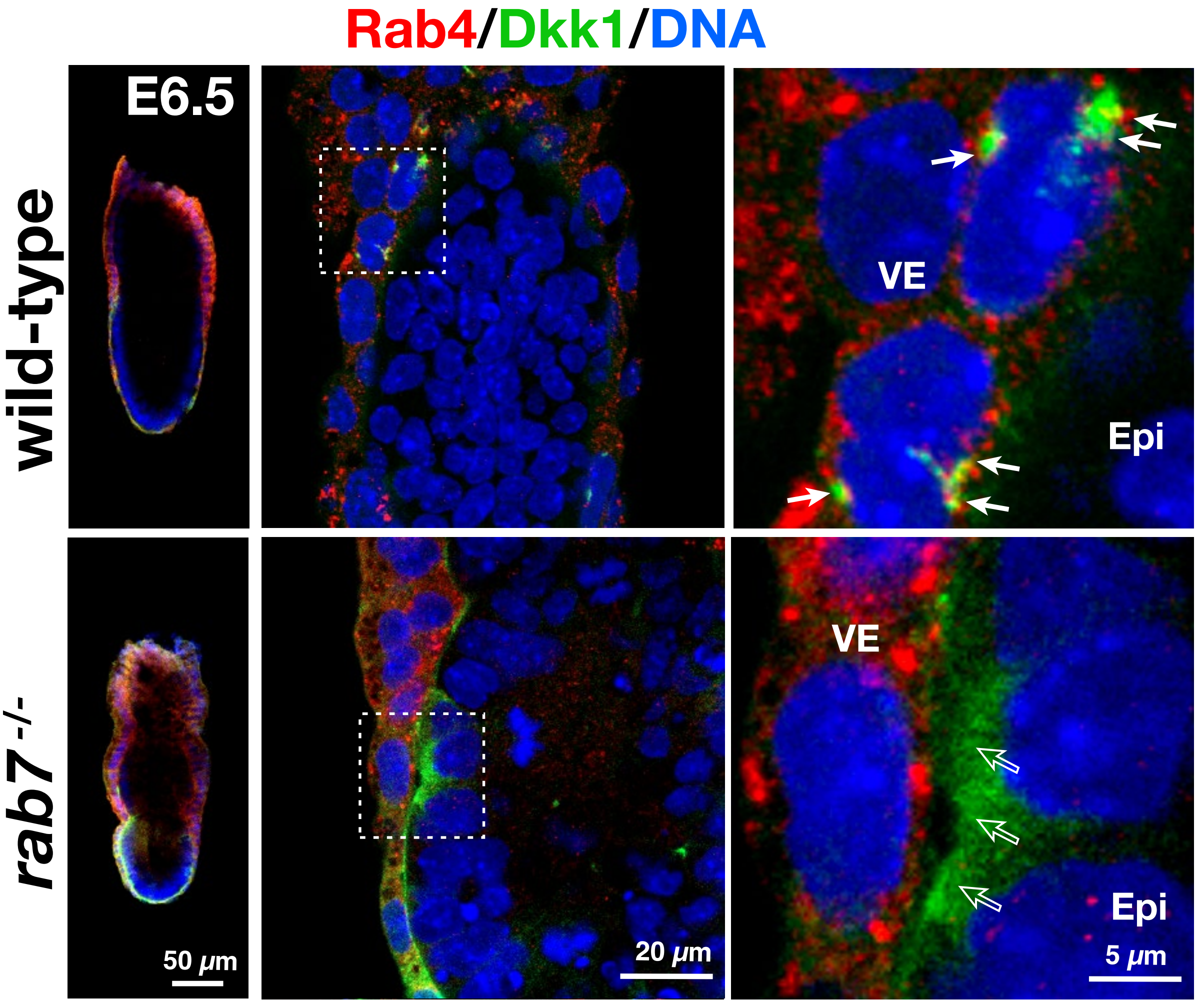

Figure S3: Dkk1 trafficking occurred at endocytic compartments

E6.5 wild-type (6 embryos) and Rab7-deficient embryos (3 embryos) stained for Dkk1 (green) and Rab4 (red) and images were obtained by confocal microscopy. The areas outlined by white dashed lines are magnified. Arrows indicated the Dkk1 signals superimposed with those of Rab4 in the visceral endoderm cells of wild-type embryos. Outlined arrows indicated the dot-like or diffused Dkk1 signals observed in Rab7-deficient embryos.
